# Epigenetic silencing of miR-375 induces trastuzumab resistance in HER2-positive breast cancer by targeting IGF1R

**DOI:** 10.1186/1471-2407-14-134

**Published:** 2014-02-26

**Authors:** Xing-Ming Ye, Hua-Yu Zhu, Wen-Dong Bai, Ting Wang, Lei Wang, Ying Chen, An-Gang Yang, Lin-Tao Jia

**Affiliations:** 1 State Key Laboratory of Cancer Biology, Department of Biochemistry and Molecular Biology, Fourth Military Medical University, Xi’an 710032, China; 2 Fujian Provincial Cancer Hospital, the Teaching Hospital of Fujian Medical University, Fuzhou 350014, China; 3 Department of Immunology, Fourth Military Medical University, Xi’an 710032, China

**Keywords:** miR-375, Insulin-like growth factor 1 receptor, Trastuzumab resistance, erbB2/HER2, Breast cancer

## Abstract

**Background:**

Resistance to humanized monoclonal erbB2/HER2 antibody, trastuzumab (Herceptin), has become a pivotal obstacle for targeted therapy of HER2-positive breast cancers. The activation of alternative growth factor receptors, in particular, the insulin-like growth factor 1 receptor (IGF1R), represents a common feature of trastuzumab-refractory cells; however, the underlying mechanism remains elusive.

**Methods:**

Trastuzumab-resistant breast cancer SKBr-3 cells were generated by long-term in vitro culture of SKBr-3 cells in the presence of trastuzumab. Among the differentially expressed microRNAs (miRNAs) screened by microarray analysis, candidate miRNA(s) predicted to target IGF1R was studied for its role in conferring trastuzumab resistance. The mechanism underlying decreased expression of IGF1R-targeted miRNA in refractory cells was also addressed.

**Results:**

miR-375, which was downregulated and predicted to target IGF1R in trastuzumab-resistant HER2-positive breast cancer cells, could indeed inhibit the cellular luciferase activity in a reporter construct containing the 3′-UTR of IGF1R. Overexpression of miR-375 restored the sensitivity of cells to trastuzumab, while inhibition of miR-375 conferred trastuzumab resistance on HER2-positive breast cancer cells. Blockade of DNA methylation and histone deacetylation restored the expression of miR-375 in trastuzumab-resistant cells. A reverse correlation between the levels of miR-375 and IGF1R was validated in clinical breast cancers.

**Conclusions:**

Epigenetic silencing of miR-375 causes the upregulation of IGF1R, which at least partially underlies trastuzumab resistance of breast cancer cells. Our study has implications for miR-375 as a potential target in combination with trastuzumab for treating HER2-positive breast cancers.

## Background

Resistance to prevalent anticancer drugs is a hallmark of advanced breast cancers that causes mortality in the majority of patients by facilitating cancer progression and distant metastasis [1,2]. Overexpression of the *ERBB2* gene, which encodes the oncoprotein HER2, occurs in 20 to 25% of human breast cancers and is associated with poor prognosis. The humanized anti-HER2 antibody, trastuzumab (Herceptin), has been successfully used for the treatment of HER2-positive early stage and metastatic breast cancers [3-5]. However, the response rate of patients with HER2-positive breast cancers to trastuzumab monotherapy is less than 35%, and this rate is only slightly increased (to approximately 40%) when trastuzumab is combined with microtubule-stabilizing drugs [5,6]. Furthermore, most patients that respond to the initial trastuzumab treatment develop resistance within a year [5]; therefore, clarifying the mechanisms underlying trastuzumab resistance will provide great impetus for the development of novel strategies for breast cancer therapy [7].

Various mechanisms have been reported to cause resistance of breast cancers to trastuzumab, including reduced HER2 expression or antibody affinity, increased pro-survival signaling through alternative receptor tyrosine kinases, and altered intracellular signaling such as the loss of PTEN expression, reduced activity of cell cycle regulator p27kip1, or increased Akt activity, which result in the over-proliferation of cells [8,9]. In particular, insulin-like growth factor-1 receptor (IGF1R) is thought to play a key role in the acquisition of cancer resistance to trastuzumab and other targeted pharmaceuticals [10,11]; however, little is currently known regarding the regulation of IGF1R in these cells during the development of resistance to trastuzumab.

MicroRNAs (miRNAs) are a class of short, non-coding RNAs that regulate gene expression by specifically degrading mRNAs or causing translational repression. It is well-documented that miRNAs play crucial roles in modulating multiple pathways responsible for cancer progression. These miRNAs are either pro-oncogenic, by targeting tumor suppressor genes, or tumor suppressive, by silencing the oncogenes [12]. In this study, microarray-based miRNA profiling was used to screen for miRNAs that respond to trastuzumab treatment. miR-375 was among the few miRNAs significantly downregulated in breast cancer cells treated with trastuzumab. This miRNA was found to target IGF1R and was identified as the key regulator of trastuzumab responsiveness via targeting IGF1R. Ectopic expression of miR-375 inhibited IGF1R expression and restored sensitivity of breast cancer cells to trastuzumab. These data suggest that miR-375 may be a novel therapeutic targets for trastuzumab-resistant breast cancers.

## Methods

### Cell culture and generation of trastuzumab-resistant cells

The human breast cancer SKBr-3 and human embryonic kidney 293 (HEK293) cell lines were obtained from the Institute of Biochemistry and Cell Biology, Chinese Academy of Sciences. SKBr-3 cells were cultured in RPMI 1640 media supplemented with 10% fetal bovine serum (FBS) and HEK293 cells were cultured in D-MEM high glucose medium containing 10% FBS. Both cell lines were maintained at 37°C in a humidified atmosphere containing 5% CO_2_. Trastuzumab/Herceptin (Roche, Basel, Switzerland) was dissolved in sterile water. Trastuzumab-resistant cells were developed by continuous culture of SKBr-3 cells in the presence of 5 μg/ml trastuzumab for 6 months, as reported previously [13]. Thereafter, trastuzumab-resistant and parental SKBr-3 cells were cultured with or without trastuzumab, respectively.

### Plasmid construction and preparation of lentivirus

Short hairpin RNAs (58 nt) were designed to target 21 nt sequences of *IGF1R* mRNA and *GFP* mRNA as a control. These sequences were subjected to BLAST query to confirm the lack of homology to other known genes. The shRNA targeted sequences were as follows: *IGF1R*, 5′-GCTGATGTGTACGTTCCTGAT-3′; *GFP*, 5′-CGTGATCTTCACCGACA AGAT-3′. Paired deoxyribonucleotide oligos encoding the shRNAs were synthesized, annealed, and cloned into the *Eco*RI and *Nco*I sites of the pLKO.1 vector (Addgene, USA). Lentivirus packaging and infection were performed according to standard protocols as recommended by the manufacturer.

The sequences of the primers used for PCR amplification of the pre-miR-375 coding sequence were as follows: 5′-CGGAATTCAGGGTGGCTGGGAAAGG-3′ and 5′-CCGCTCGAGCCGTATTACGACGCAGAATG-3′. The resulting PCR fragment was cloned into the pMD-18 T vector (Takara, Japan) and successful cloning was confirmed by DNA sequencing. The pre-miR-375 coding sequence was then subcloned into the lentivirus-based expression plasmid pLenti6/V5 (Invitrogen, USA), and virus packaging and infection were performed according to protocols as recommended by the manufacturer.

The miR-375 mimics, miR-375 inhibitor, and negative controls were purchased from Shanghai Genechem Inc. (Shanghai, China). Transfection of cells with 50 nM of each miRNA was performed using Lipofectamine 2000 reagent (Invitrogen, Carlsbad, CA), according to the manufacturer’s instructions.

### Colony formation assay

Colony formation in soft agar was tested by plating 1 × 10^4^ parental and trastuzumab-resistant SKBr-3 cells in 0.4 ml of complete DMEM medium supplemented with 0.3% low-melting temperature agarose (Seaplaque) in 12-well plates coated with 0.8 ml 0.6% low-melting temperature agarose. Colony formation of cells was monitored for 7 days in 37°C incubator and colony number and size was recorded for comparison, and microphotographed by Axiovert 40 CFL microscope on day 7. For plate colony formation assay, suspensions of cells were inoculated in 6-well flat-bottomed plates with a density of 2000 cells per well. Cells were dispersed evenly by slightly shaking the plates and were then incubated in complete DMEM medium supplemented with 5 μg/ml trastuzumab at 37°C and 5% CO_2_ for 7 days, until the visible clones appeared. Plates were then gently washed and subjected to Giemsa staining. Viable colonies containing at least 50 cells were counted. All experiments were repeated in triplicate and the average values were presented.

### Luciferase reporter assay

**Figure 1:**
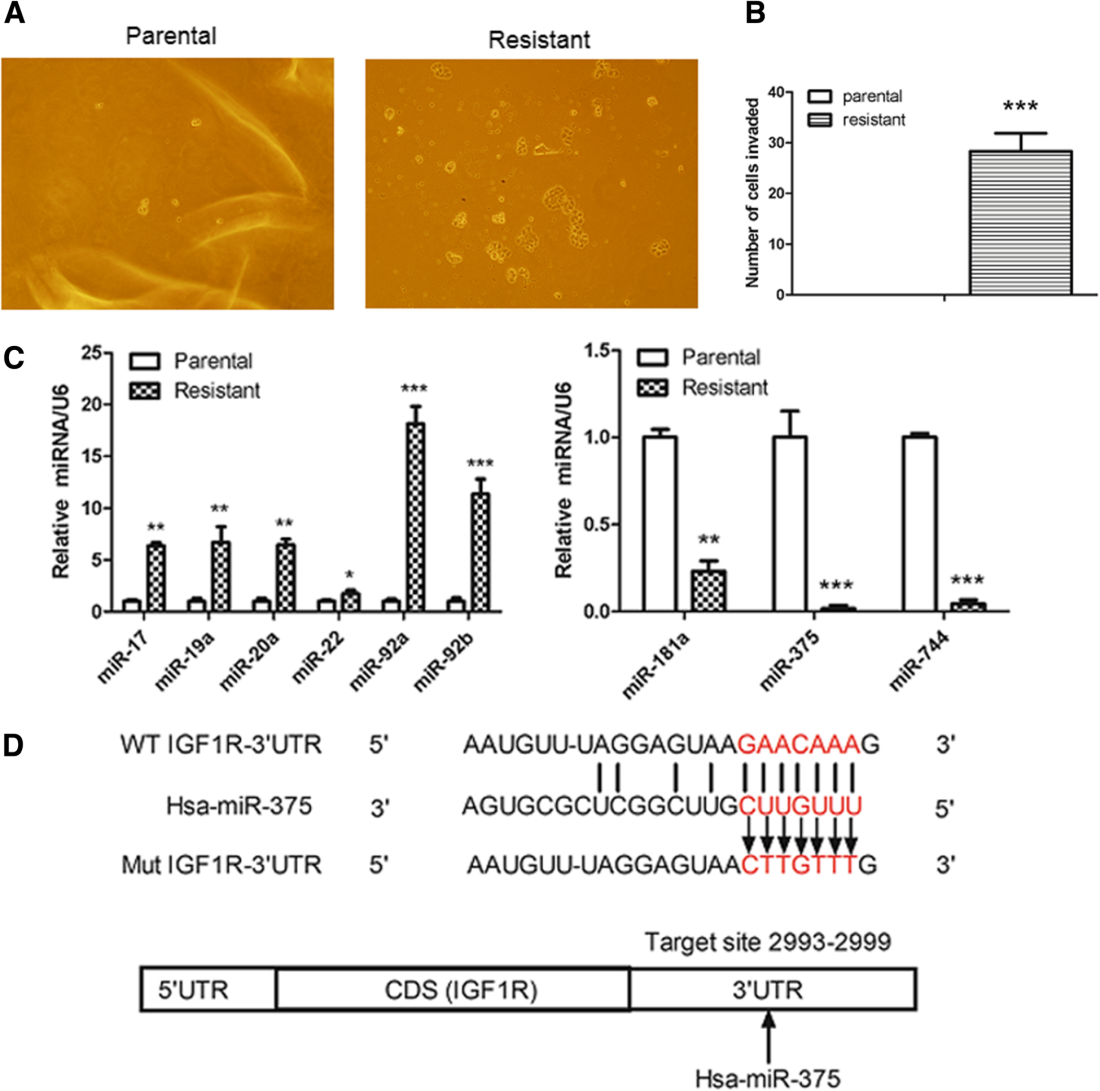
**Trastuzumab-resistant and parental breast cancer SKBr-3 cells display distinct characteristics and miRNA expression profiles. A**. SKBr-3 cells were cultured in the presence of trastuzumab (5 μg/ml) for 6 months to obtain resistant cells. A colony formation assay in soft agar was performed using parental and resistant cells. The images are representative of triplicate experiments on day 7 after cell seeding. **B**. MTT assays of parental and trastuzumab-resistant SKBr-3 cells in the absence of trastuzumab. **C**. Confirmation of the differential expression of the indicated miRNAs by qRT-PCR. Each assay was performed in triplicate and the expression levels of each miRNA were normalized to those of snRNA RNU6B (U6). **D**. The predicted human miR-375 binding site at nucleotides 2993-2999 of the 3′ UTR of wild-type (WT) IGF1R (sense). The sequence of the pGL3-IGF1R-mut construct containing mutated nucleotides at the miR-375 binding site within the 3′ UTR of IGF1R is also shown. All data are represented as the mean ± SD or are representative of n = 3 replicates. *P <0.05, **P <0.01 and ***P <0.001.

The 3′UTR of the wild-type *IGF1R* and a variant containing mutations in the putative miR-375 binding site (Figure 1D) were inserted downstream of the firefly luciferase gene in the pGL3 vector (Promega, Madison, USA). Primers used for PCR amplification of the *IGF1R* 3′UTR were as follows: wild-type, 5′-TTTGAATTCTTCAATCACTGTAGAAAAGCCCCAT-3′ and 5′-TTTCTGCAGAAGGGGAGCAAACCCAATGC-3′; mutant, 5′-GGCTGGTTGTCTTTGCTTGTTTCTGCTTCTGTGCAG-3′ and 5′-CTGCACAGAA GCAGAAACAAGCAAAGACAACCAGCC-3′. Trastuzumab-resistant SKBr-3 cells were co-transfected with reporter constructs, an internal control vector (pGL4.73), and synthetic miR-375 mimics. Forty-eight hours after transfection, cells were rinsed with phosphate buffered saline (PBS), and then luciferase activity was assayed using the Dual-Luciferase Reporter Assay System (Promega, USA) and a luminometer. The luciferase activity of each lysate was normalized to the activity of Renilla luciferase driven by the constitutively expressing promoter in the phRL vector. Basal promoter activity was measured as the fold change relative to the activity observed with the basic pGL3 vector alone.

### Quantitative RT-PCR for miRNAs and protein-coding genes

Total RNA was extracted from each cell line using TRIzol reagent (Invitrogen, USA) according to the manufacturer’s protocol. Reverse transcription was performed using SuperScriptTM II Reverse Transcriptase (Invitrogen, USA), and cDNAs were amplified and detected using SYBR@ Premix Ex TaqTM (TaKaRa, Japan). To quantify miRNAs, total RNA was reversed transcribed using the miScript Reverse Transcription Kit (Qiagen, Germany) and then amplified using SYBR@ Premix Ex TaqTM (Takara, Japan). GAPDH and U6 RNA were used as internal loading controls for mRNAs and miRNAs, respectively. The following primers were used for PCR amplification: a universal primer (UP) provided with the miScript Reverse Transcription Kit and 5′-TTTGTTCGTTCGGCTCGCGTGA-3′ or 5′-GTGCTCGCTTCGGCAGCACATAT-3′ for miR-375 or U6 RNA, respectively; 5′-GGACAGGTCAGAGGGTTTC-3′ and 5′-CTCGTAACTCTTCTCTGTGCC-3′ for IGF1R; and 5′-GCCCAATACGACCAAATCC-3′ and 5′-AGCCACATCGCTCAG ACAC-3′ for GAPDH.

### Proliferation assay

Cell proliferation was measured using the MTT [3-(4,5-dimethylthiazol-2-yl)-2,5- diphenyl-2*H*-tetrazolium bromide] assay as described previously with minor modifications [14]. Briefly, cells were seeded into 96-well plates at a density of 3000 cells per well, and were incubated with pre-miRNA lentiviruses. 5 μg/ml trastuzumab were added into the medium 24 h later, and the medium was replaced by 100 μl fresh serum-free medium containing 0.5 g/l MTT 24 h after addition of trastuzumab. After incubation at 37°C for 4 h, the MTT medium was removed by aspiration and 50 μl of DMSO was added to each well. After incubation at 37°C for a further 10 min, the A490 value of each sample was measured using a plate reader.

### Western blotting analysis

Cells were starved in serum-free medium for six hours, and were switched to culture in complete medium for 10 min. Cells were then washed with PBS twice and then proteins were extracted, separated on an SDS/PAGE gel, transferred onto PVDF membrane, and subjected to immunoblot analyses. Blotting was performed using antibodies targeting IGF1R, AKT (Cell Signaling Technology, USA), phosphorylated AKT (Epitomics, USA), and cyclin D1 (Santa Cruz, USA). Goat anti-rabbit and goat anti-mouse immunoglobulin horseradish peroxidase-linked F(ab)_2_ fragments (ZB-2305, Zhong Shan Jin Qiao, China) were used as secondary antibodies.

### Apoptosis assay

Cells were plated into 6-well plates at a density of 4 × 10^5^ cells per well, and were incubated with pre-miRNA lentiviruses or transfected with miRNA antisense using Lipofectamine 2000 reagent (Invitrogen, USA). 24 h later, SKBr-3 cells were treated with trastuzumab in a final concentration of 5 (parental cells) and 10 μg/ml (trastuzumab-resistant cells), respectively. After 24 h exposure to trastuzumab, cells were stained with Annexin V-FITC and propidium iodide (PI), and then flow cytometry was performed to detect apoptosis of the transfected cells.

### Analysis of epigenetic modifications of the miR-375 gene

Cells were treated with 5-Aza-CdR (5 μmol/L) for 3 days and/or TSA (100 μmol/L) for 24 hrs. For chromatin immunoprecipitation assay, cells were cross-linked with formaldehyde, and chromatin was fragmented by sonication. Precleared chromatin was overnight immunoprecipitated with antibodies against histone H3 (Abcam, UK) or acetylated H3K9/K14 (Upstate, USA) antibodies. The enrichment of specific DNA fragments was analyzed by PCR. The primers used for amplification of the miR-375 promoter region are as follows: 5′-CGGTGATCTCCTGGTCCTGGTCTT-3′ and 5′-AGCTTCTGTCCTCTGCTTCTCGGCT-3′, which were designed according a previous report that the 768 bp upstream of pre-miR-375 coding sequence contains the functional promoter of pri-miR-375 [15]. For bisulfite modification and promoter methylation analysis, genomic DNA was treated by bisulfite and then studied by methylation-specific PCR (MSP) as previously described to detect the methylation of 2 CpGs 60 bp upstream of the pre-miR-375 coding sequence [16]. MSP primers are as follows: 5′-CTGGACAGGTGTGAGTGTGTGTGTCTG-3′, and 5′-TGATCTCCTGGTCCTGGTCTTCCGCG-3′ for methylated allele or 5′-TGATCTCCTGGTCCTGGTCTTCCACA -3′ for unmethylated allele.

### In vivo tumor growth and mouse survival assay

Four- to six-week-old BALB/c nude mice (Institute of Zoology, Chinese Academy of Sciences) were randomly grouped to monitor tumor growth and mouse survival, respectively. 3 × 10^6^ cells were injected into the right mammary fat pad of each mouse. After 2 weeks, mice were intravenously injected with 10 mg/kg trastuzumab twice a week. For tumor growth assay, tumor volume was calculated as follows: tumor volume = width^2^ × length/2. Mice were sacrificed 45 days post-first trastuzumab injection, and tumors were seperated for weighing. For survival assay, the survival of mice in each group was recorded, and the ratios of surviving mice were plotted. All experimental protocols were performed in accordance with the ARRIVE (Animal Research: Reporting In Vivo Experiments) guideline of the UK and were approved by the Institutional Animal Care and Use Committee of Fourth Military Medical University.

### Clinical sample collection

Breast cancer samples were collected from breast cancer patients, aged from 26 to 70, with informed consent at Xijing Hospital, the Fourth Military Medical University, Xi’an, China. 17 of 40 samples were confirmed HER2-positive (immunohistochemistry score 3 or fluorescence in-situ hybridisation positive). The tissues were immediately frozen in liquid nitrogen and stored at -80°C until use. Samples collection was approved by the Ethics Committee of the Fourth Military Medical University.

### Statistical analysis

Statistical analyses were performed using SPSS version 16.0 for Windows. Student’s *t* tests were used to analyze the results expressed as the mean ± SD. The chi-squared test or Fisher’s exact test was used to analyze the association between the expression levels of miR-375 and IGF1R. The survival curves were plotted after Kaplan-Meier analysis. Differences were considered significant when the *P*-value was less than 0.05.

## Results

### Trastuzumab-resistant breast cancer cells exhibit survival or proliferation advantages over parental cells

Human breast cancer SKBr-3 cells, which overexpress HER2, were cultured continuously for 6 months in the presence of 5 μg/ml trastuzumab, resulting in the acquisition of trastuzumab resistance in the surviving cell population [17]. Compared with the parental cells, the resistant SKBr-3 cells displayed dramatically increased colony formation on the agar plates (Figure 1A) and had a significantly higher viability or proliferative capacity in an MTT assay (*p <* 0.05, Figure 1B). These results suggest that trastuzumab-resistant HER2-positive breast cancer cells exhibit anchorage-independent growth and proliferation advantages *in vitro* over non-resistant cells.

### Distinct miRNA expression profiles in parental and trastuzumab-resistant cells

To investigate the roles of miRNAs in the resistance of breast cancers to trastuzumab, a microarray analysis of miRNA profiles in trastuzumab-resistant and parental SKBr-3 cells was previously performed using miRCURY LNA arrays. Using a cutoff greater than a two-fold differences in miRNA expression, we identified differentially expressed miRNAs in trastuzumab-resistant cells compared with the parental cells, which was already submitted to the Gene Expression Omnibus (GEO, assigned accession #: GSE47011). Differential expression between parental and trastuzumab-resistant SKBr-3 cells was confirmed for nine of these miRNAs by quantitative RT-PCR (qRT-PCR). Following the acquisition of trastuzumab resistance, expression levels of miR-17, miR-19a, miR-20a, miR-22, miR-92a, and miR-92b were significantly upregulated; and expression levels of miR-181a, miR-375, and miR-744 were significantly downregulated (*p <* 0.001, Figure 1C). The potential target genes of these miRNAs were then predicted using the well-documented software programs like PicTar, TargetScan and miRanda (data not shown), followed by a functional clustering analysis classified by the MicroCosm Targets program (EMBL-EBI) [18,19]. Among the differentially expressed miRNAs, we focused particularly on miR-375, which showed the second largest absolute fold change in the microarray analysis (*p* < 0.001), because this miRNA was predicted to target IGF1R, a receptor tyrosine kinase dominantly upregulated in trastuzumab-resistant cells (Figure 1D) [13].

### miR-375 modulates trastuzumab resistance in breast cancer cells

**Figure 2:**
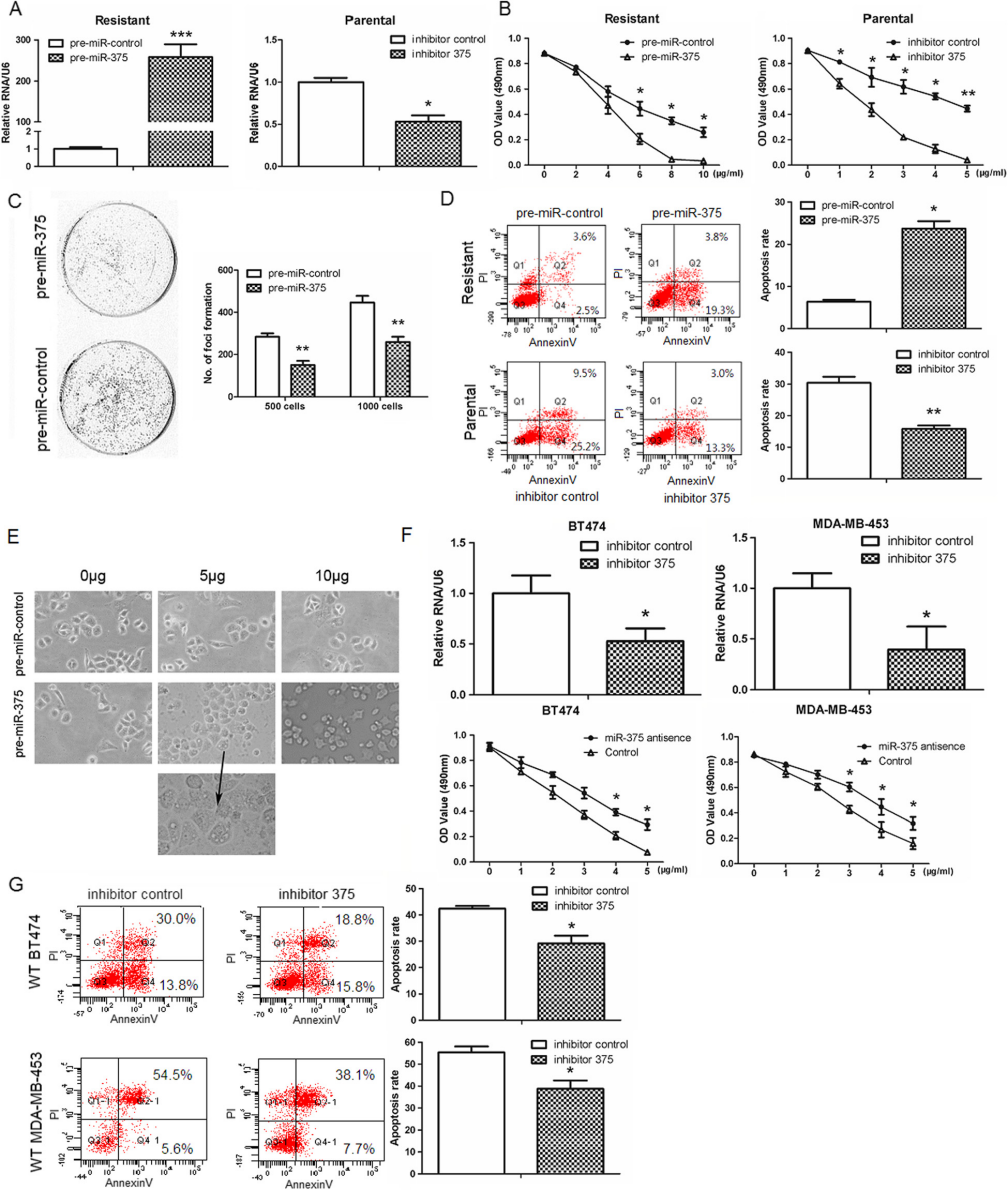
**miR-375 modulates trastuzumab resistance in breast cancer cells.** Trastuzumab-resistant SKBr-3 cells were infected with lentiviral vectors expressing pre-miR-control or pre-miR-375, and parental cells were transfected with a control RNA or a miR-375-specific inhibitor. **A**. qRT-PCR analyses of miR-375 expression. Data were normalized to mock-transfected cells and the expression levels of miR-375 were normalized to U6. **B.** MTT assays of modified cells after treatment with increasing concentrations of trastuzumab. **C**. Plate colony formation assays of modified trastuzumab-resistant cells in the presence of trastuzumab (5 μg/ml). **D**. Flow cytometry analyses of modified cells. Resistant and parental cells were treated with 10 μg/ml or 5 μg/ml trastuzumab, respectively, for 24 h, and then stained with FITC-conjugated Annexin V and PI. **E**. Microscopy images of modified trastuzumab-resistant SKBr-3 cells. Cells were treated with the indicated concentrations of trastuzumab for 24 h and images were captured using a phase contrast microscope. The arrow indicates autophagosome-like bodies. **F**. Upper panel: qRT-PCR assays were performed to identify the expression levels of miR-375 in human breast cancer BT474 and MDA-MB-453 cells. Lower panel: MTT assay of cells transfected with control or miR-375 antisense RNA and treated with various concentrations of trastuzumab for 24 h. **G**. Flow cytometry analyses of apoptosis in cells transfected with control or miR-375 antisense RNA. Twenty-four hours post-transfection, cells were treated with trastuzumab (5 μg/mL) for another 24 h, and cells were stained with FITC-conjugated Annexin V and PI. All data are represented as the mean ± SD or are representative of n = 3 replicates. *P <0.05, **P <0.01 and ***P <0.001.

To further investigate the role of miR-375 in trastuzumab resistance of breast cancers, the levels of miR-375 were altered in both parental and trastuzumab-resistant cells by lentivirus-delivered pre-miR-375 and introduction of miR-375 antisense RNA. Compared with cells expressing a control pre-miRNA, trastuzumab-resistant cells expressing pre-miR-375 displayed significantly higher cellular levels of miR-375 and enhanced sensitivity to trastuzumab (*p* < 0.05, Figure 2A and B). Conversely, transfection of parental SKBr-3 cells with miR-375 antisense RNA caused a significant decrease in miR-375 expression, and conferred resistance of these cells to trastuzumab (*p* < 0.01, Figure 2A and B). Overexpression of pre-miR-375 also significantly suppressed in vitro colony formation by trastuzumab-resistant cells (*p* < 0.01, Figure 2C). We then examined the apoptosis of cells after treatment with trastuzumab for 24 h. Pre-miR-375 overexpression caused a significant increase in apoptosis of trastuzumab-resistant cells (*p* < 0.05, Figure 2D), and inhibition of miR-375 significantly suppressed apoptosis of parental SKBr-3 cells (*p* < 0.01, Figure 2D). In the presence of trastuzumab, overexpression of pre-miR-375 consistently induced a conversion from a healthy and mitotic morphology to a phenotype with shrinking and granulated cytoplasm (Figure 2E). The role of miR-375 in the responses of other HER2-positive breast cancer cell lines to trastuzumab was then investigated. Inhibition of miR-375 by a specific antisense RNA promoted survival of both BT474 and MBA-MD-453 cells in the presence of trastuzumab (*p* < 0.05, Figure 2F and G). These data indicate that loss of miR-375 expression is critically involved in the development of trastuzumab resistance in breast cancer cells.

### miR-375 directly targets IGF1R in breast cancer cells

**Figure 3:**
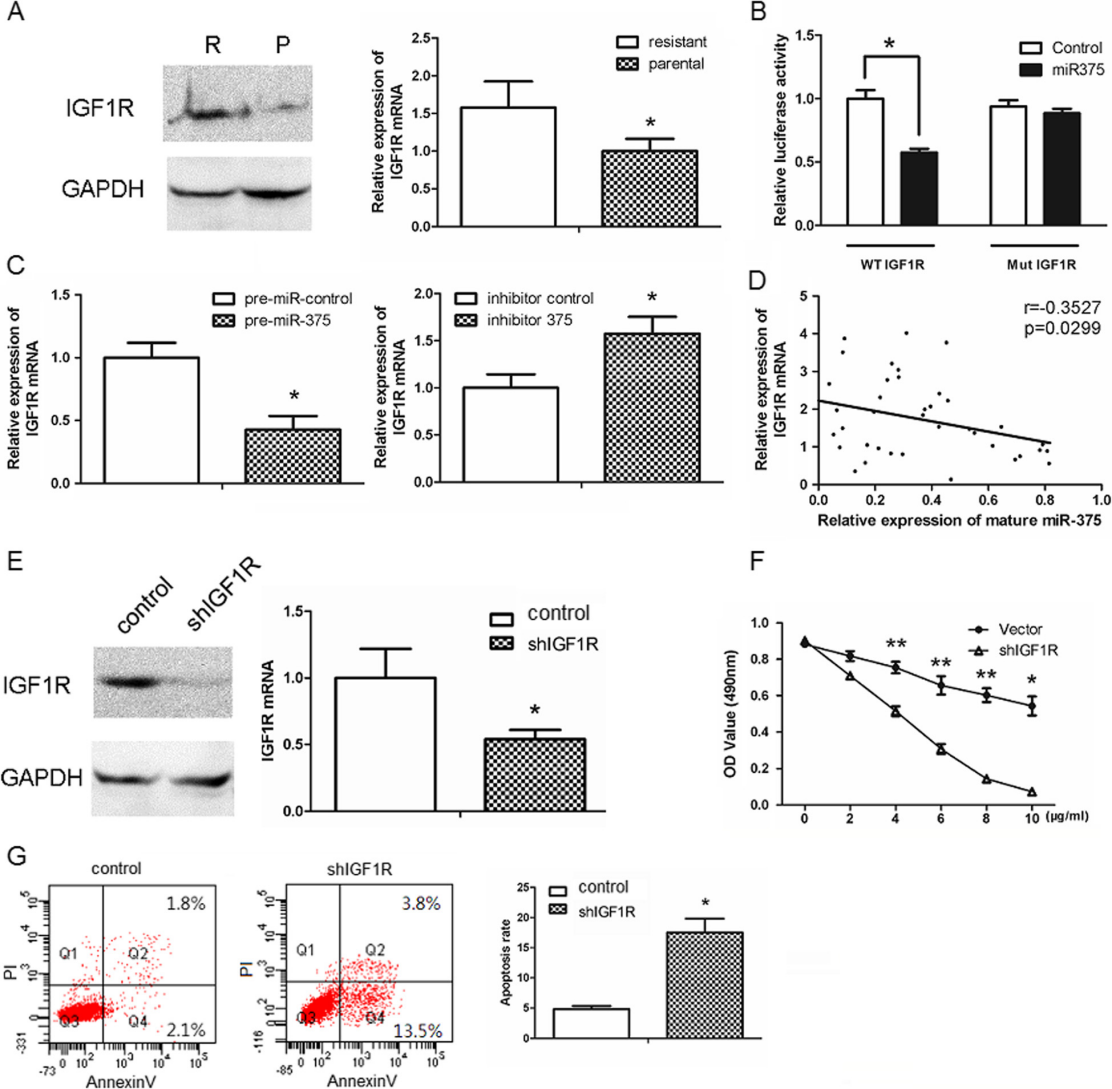
**miR-375 restores trastuzumab sensitivity by directly targeting IGF1R in breast cancer cell. A**. Western blot (left) and qRT-PCR (right) analyses of IGF1R expression in parental (P) and trastuzumab-resistant (R) SKBr-3 cells. The qRT-PCR data were normalized to GAPDH. **B**. Luciferase activity measured 24 h after co-transfection of trastuzumab-resistant SKBr-3 with pGL3 constructs containing the wild-type or mutant 3′UTR of IGF1R, an internal control vector (pGL4.73), and synthetic miR-375 mimics. Data were normalized to the luciferase activity of control (vehicle transfected) cells. **C**. qRT-PCR analyses of IGF1R expression in SKBr-3 cells as modified in Figure 2. Data were normalized to mock-transfected cells. **D**. Pearson’s correlation analysis of the relative expression level of miR-375 (normalized to U6) and IGF1R mRNA (normalized to GAPDH) as determined using qRT-PCR in 40 human breast cancer tissue samples. **E**. Western blot and qRT-PCR analyses of trastuzumab-resistant SKBr-3 cells infected with a lentivirus vector expressing GFP- (control) or IGF1R-specific shRNA. Data were normalized to those of GAPDH. **F**. MTT assays of cells described in **(E)** after treatment with the indicated concentration of trastuzumab for 24 h prior to analysis. **G**. Flow cytometry analyses of cells in **(E)**. Cells were treated with trastuzumab (10 μg/ml or 5 μg/ml) for 24 h and then stained with Annexin V and PI. All data are represented as the mean ± SD of n = 3 replicates. *P <0.05 and **P <0.01.

Next, we investigated whether miR-375 suppresses trastuzumab resistance by targeting IGF1R. In contrast to the correlation of decreased miR-375 with trastuzumab resistance, IGF1R protein and mRNA levels were higher in trastuzumab-resistant cells than parental SKBr-3 cells (Figure 3A). A 200 bp region of the 3′ UTR of IGF1R containing the potential miR-375 binding site was then investigated using a firefly luciferase reporter assay. Compared with cells transfected with a control pre-miRNA, luciferase activity was reduced by approximately 40% in cells expressing miR-375 (*p* < 0.05, Figure 3B). However, activity of the firefly luciferase gene under the control of the IGF1R 3′ UTR containing mutations in the putative miR-375 binding site was not affected by overexpression of miR-375 (*p* < 0.05, Figure 3B). Consistent with these results, overexpression of pre-miR-375 in trastuzumab-resistant SKBr-3 cells resulted in a significant reduction in IGF1R mRNA levels, while inhibition of miR-375 in parental SKBr-3 cells resulted in upregulation of IGF1R mRNA (Figure 3C). In clinical breast cancer samples, miR-375 expression was inversely correlated with IGF1R mRNA levels (*p* = 0.0299, Figure 3D). These data suggest that IGF1R is a direct target of miR-375 in breast cancer cells.

### Suppression of IGF1R inhibits trastuzumab resistance of breast cancer cells

To further examine the role of IGF1R in trastuzumab resistance of breast cancer cells, IGF1R was knocked down in trastuzumab-resistant cells using a short hairpin RNAs (shRNAs) (Figure 3E). Similar to the effects of miR-375 overexpression, silencing of IGF1R partially restored the sensitivity of SKBr-3 cells to trastuzumab (*p* < 0.01, Figure 3F and G), suggesting that IGF1R, as a target gene of miR-375, is critically involved in trastuzumab resistance of breast cancers.

### Overexpression of miR-375 partially restores trastuzumab sensitivity in vivo

**Figure 4:**
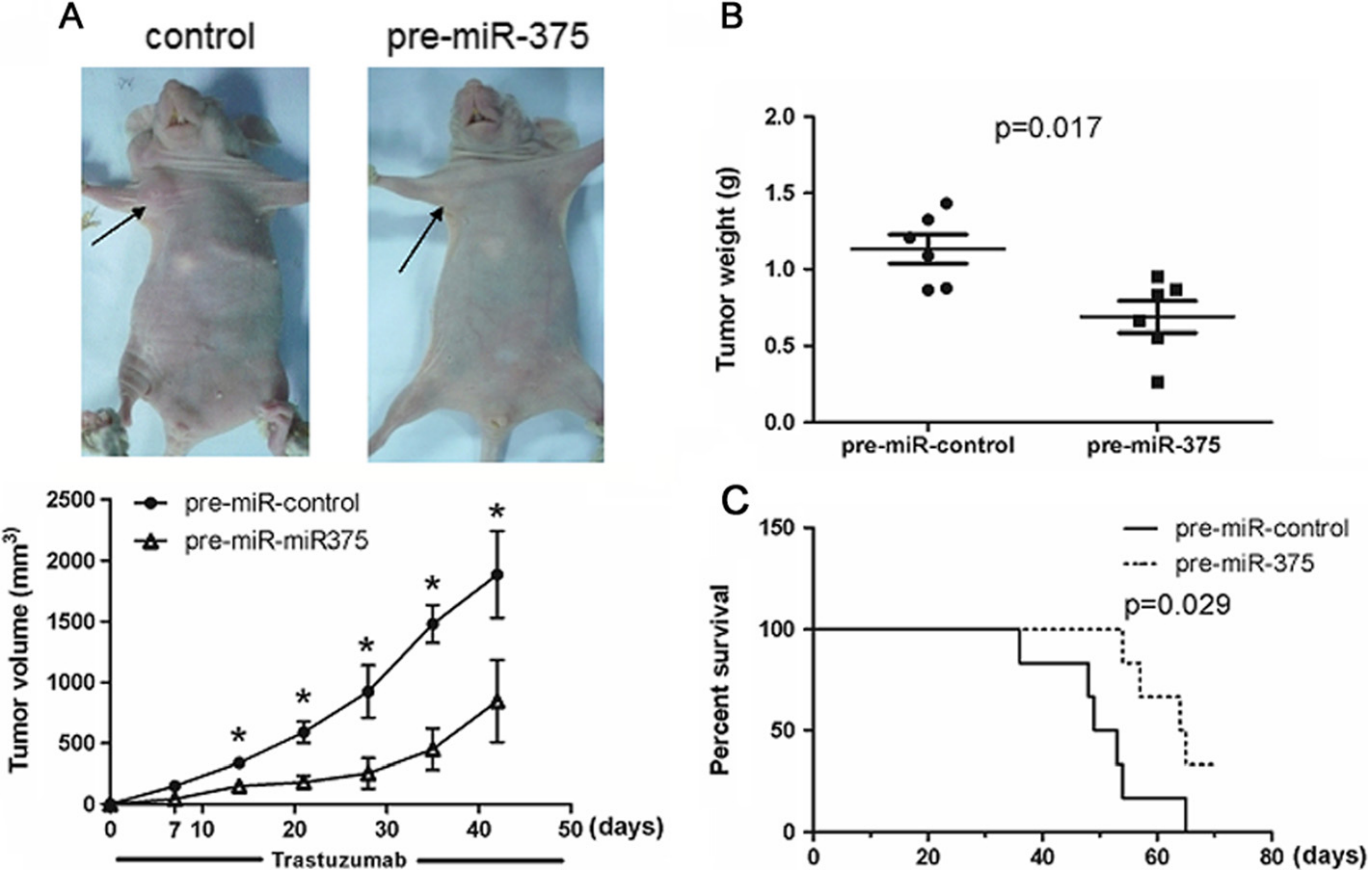
**miR-375 modulates trastuzumab resistance of HER2-positive breast cancer xenografts.** Nude mice were inoculated in the mammary fat pad with trastuzumab-resistant SKBr-3 cells overexpressing pre-miR-375 or control pre-miRNA to allow tumor development. Mice were intravenously injected with 10 mg/kg trastuzumab twice a week. **A**. Tumor volume in the trastuzumab-treated mice. Data are represented as the mean ± SD of six mice. *P <0.05. **B**. Tumor weight at the end of the treatment period (45 days after first trastuzumab injection). The bars represent the mean ± SD of six mice. **C**. Kaplan-Meier survival curves of the trastuzumab-treated mice (n = 6).

To investigate whether miR-375 can reverse the resistance of HER2-positive breast cancers to trastuzumab in vivo, xenograft models were generated using trastuzumab-resistant SKBr-3 cells modified to overexpress pre-miR-375 or control pre-miRNA. These cells were injected into the mammary fat pads of athymic nude mice, and then the mice were intravenously injected with 10 mg/kg trastuzumab twice a week [13]. Compared with mice bearing tumors derived from SKBr-3 cells expressing a control pre-miRNA, mice inoculated with pre-miR-375-modified SKBr-3 cells displayed significantly suppressed tumor development and growth (*p* < 0.05, Figure 4A and B). A Kaplan-Meier survival analysis showed prolonged survival of mice challenged with SKBr-3 cells expressing pre-miR-375, compared with those inoculated with the control cells after treatment with trastuzumab (*p* = 0.029*,* Figure 4C). These data suggest that the overexpression of miR-375 may sensitize trastuzumab-resistant breast cancers to trastuzumab in vivo.

### Epigenetic mechanisms and PI3K/Akt pathway are involved in miR-375/IGF1R -mediated trastuzumab resistance

**Figure 5:**
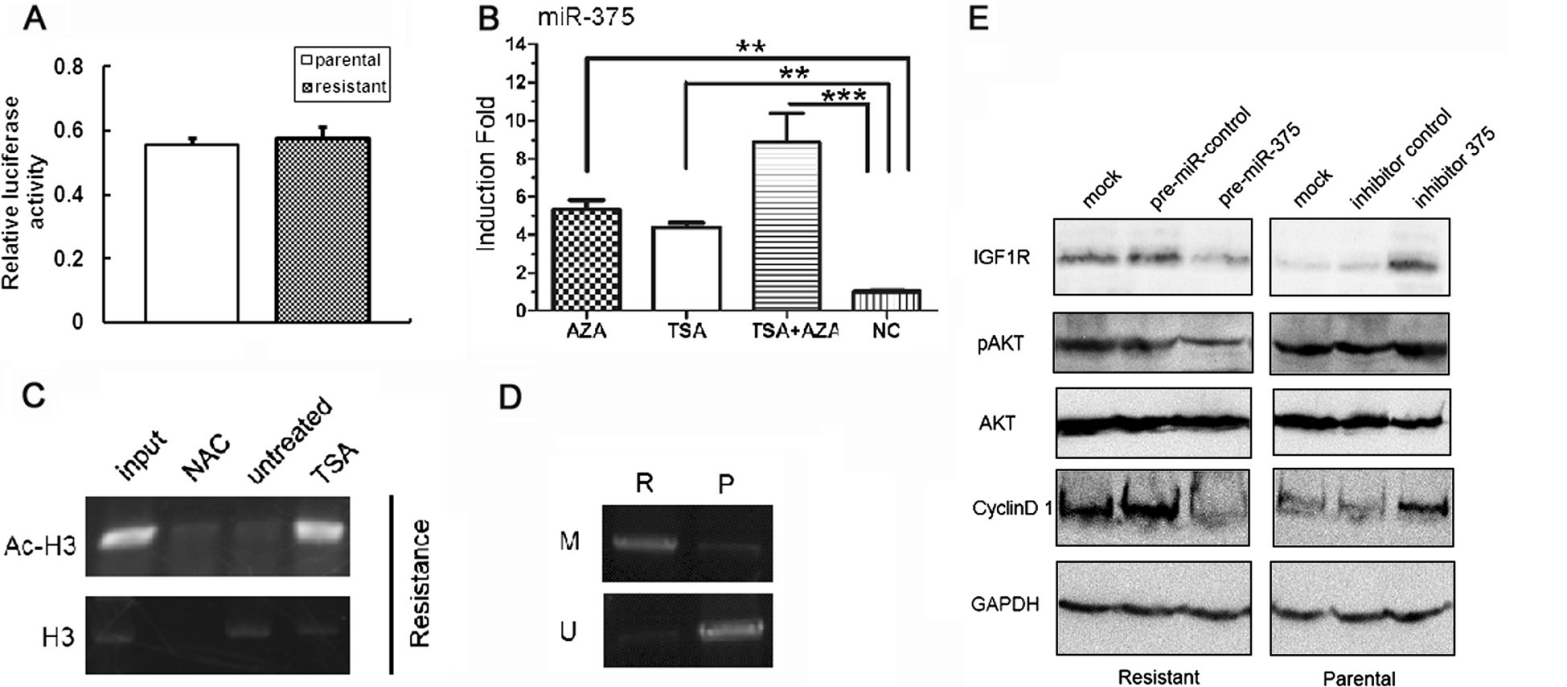
**Epigenetic mechanisms underlie miR-375 deregulation, and PI3K/Akt pathway is involved in miR-375/IGF1R-mediated trastuzumab resistance. A**. The luciferase reporter construct of miR-375 promoter was introduced into parental and trastuzumab-resistant SKBr-3 cells, followed by assays of relative cellular luciferase activity. Data were normalized to the luciferase activity of control (vehicle transfected) cells. **B**. Cells were treated with 5-Aza-CdR (5 μmol/L) for 3 days and/or TSA (100 μmol/L) for 24 hrs. qRT-PCR was performed to quantify miR-375. NC, non-treated control. **C**. The levels of acetylated histone H3K9 in the miR*-*375 promoter region were determined by chromatin immunoprecipitation assay in indicated cells. NAC, nonspecific antibody control. Ac-H3, acetylated H3. **D**. Methylation-specific PCR analysis of miR-375 promoter region in parental (P) and trastuzumab-resistant (R) SKBr-3 cells. M, methylated allele; U, unmethylated allele. **E**. Western blot analyses of trastuzumab-resistant SKBr-3 cells expressing pre-miR-375 or control pre-miRNA, and parental SKBr-3 cells transfected with miR-375 antisense RNA or control RNA. GAPDH was used as the loading control. Data are represented as the mean ± SD of n = 3 replicates for A and B. **P <0.01 and ***P <0.001.

We next probed the mechanisms underlying the suppressed expression of miR-375 in trastuzumab-resistant breast cancer cells. However, the luciferase expression under the control of a miR-375 promoter in an artificial construct were comparable in parental and trastuzumab-resistant SKBr-3 cells, suggestive of the involvement of either chromosomal modification or mechanisms other than transcriptional activation in miR-375 suppression in trastuzumab-resistant SKBr-3 cells (Figure 5A). To test whether miR-375 expression was regulated by epigenetic mechanisms, trastuzumab-resistant cells were treated with the DNA methyltransferase inhibitor, 5-Aza-2′-deoxycytidine (5-Aza-CdR), and the histone deacetylatase inhibitor, Trichostatin A (TSA). As a result, blockade of DNA methylation and/or histone deacetylation caused significant upregulation of miR-375 in trastuzumab-resistant SKBr-3 cells (Figure 5B). Chromosomal immunoprecipitation detected an increased histone H3K9 acetylation in miR-375 promoter after treatment with TSA (Figure 5C), and methylation-specific PCR validated the much higher level of DNA methylation in miR-375 promoter of trastuzumab-resistant compared with the parental SKBr-3 cells (Figure 5D), suggesting the involvement of these epigenetic modifications in the downregulation of miR-375 in trastuzumab-resistant breast cancer cells.

Trastuzumab exerts its anti-tumor effect by inhibiting AKT phosphorylation in HER2-positive breast cancer cells [20]. Consistent with previous reports that AKT is activated downstream of IGF1R signaling [21], expression of pre-miR-375 in trastuzumab-resistant cells reduced the levels of IGFR1 and phosphorylated AKT proteins (Figure 5E). The opposite effect was observed in parental cells transfected with miR-375 antisense RNA (Figure 5E). Cyclin D1, which is known to be stabilized by the PI3K/AKT pathway, displayed an expression pattern similar to that of IGF1R (Figure 5E). These results suggest that IGF1R and the AKT pathway are the downstream effectors of miR-375 that mediate trastuzumab resistance of breast cancers.

## Discussion

HER2-positive breast cancers have high rates of metastasis and recurrence and are among the most threatening pathological types of cancer [6,22,23]. Over the last 15 years, the humanized monoclonal erbB2/HER2 antibody trastuzumab (Herceptin) has been successfully used for clinical treatment of patients with HER2-positive breast cancers. Nevertheless, primary or acquired resistance to this anti-tumor antibody has become the major obstacle to its clinical efficacy [3]. Here, we demonstrate that suppressed expression of miR-375, which is a tumor suppressor targeting IGF1R, contributes to trastuzumab resistance of HER2-positive breast cancer cells.

In accordance with the multifaceted mechanisms that underlie the therapeutic efficacy of trastuzumab in breast cancers, the molecular events responsible for resistance to the drug are diverse and rely largely on crosstalk between different pathways that dictate cell survival and division [24,25]. Molecules that interfere with the accessibility of HER2, activation of downstream signaling independent of HER2, and mutation of HER2, which causes decreased antibody affinity or constitutive activation, all contribute to trastuzumab resistance in breast cancers [9,26]. However, survival or mitotic signals elicited by alternative growth factor receptors are also commonly activated in these refractory cells. In this respect, IGF1R has been studied in detail and this receptor is thought to play a key role in the development of trastuzumab resistance [24,27]. Consistent with a previous report [10], we found that inhibition of IGF1R signaling alone almost completely restored the sensitivity of HER2-positive cancer cells to trastuzumab in vitro. However, it remains unclear how IGF1R is regulated in the trastuzumab-sensitive and -refractory cells. We also established that IGF1R is a direct target of miR-375, and the loss of miR-375 expression underlies a robust upregulation of IGF1R in trastuzumab-resistant cells. The results presented here are consistent with previous reports of decreased miR-375 expression in primary esophageal squamous cell cancer [28], gastric carcinoma [29], and tamoxifen-resistant breast cancer cells [30]. In addition, we found that epigenetic mechanisms including DNA methylation and histone deacetylation are responsible for miR-375 repression in trastuzumab-resistant breast cancer cells, although additional studies are required to unravel the upstream signaling events that lead to these aberrant chromatin modifications. These findings are reminiscent of a recent report that IGF1R signaling is critically involved in the dynamic maintenance of a small population of “drug-tolerant” cells via reversible alteration of the chromatin state [11]. Therefore, it is particularly worth investigation whether the epigenetic modifications observed in trastuzumab-resistant cells are also outcomes of upstream IGF1R signaling, which will eventually form a regulatory circuit to facilitate the establishement of trastuzumab resistance in breast cancers.

miRNAs are a class of small RNAs critically involved in the regulation of gene expression [12]. By targeting oncogenes or tumor suppressors, miRNAs play divergent roles in cancer occurrence, progression, and drug resistance, and may be useful for cancer therapy by artificially counteracting the signals leading to carcinogenesis [12]. The ability of a single miRNA to simultaneously target multiple genes suggests that these small RNA species are important candidates for the regulation of cellular processes that require multiple and intersecting signaling pathways, including the development of trastuzumab resistance in breast cancers [31]. Trastuzumab resistance of breast cancers can be either cell autonomous or non-autonomous; the latter reflects an extensive interaction between cancer cells and other cells in the microenvironment, including stromal and immune cells [32-34]. Although this study identified IGF1R as a target of miR-375, and other investigations revealed that cyclin E2 and the cytoskeletal protein talin2 are targeted by miR-30b and miR-194, respectively, in the development of trastuzumab resistance, the non-autonomous mechanisms of trastuzumab resistance are to be unraveled [35,36]. It is possible that alterations in the expression levels of these miRNAs contribute to trastuzumab sensitivity by targeting additional genes that are indispensable for the acquisition of resistance in breast cancer cells *in vivo*. Whereas the role of specific miRNA(s) dominates over others in different models of trastuzumab resistance, there might be wide crosstalk between the signaling events mediated by these miRNAs [12,35,36].

## Conclusions

In this study, we established that miR-375 is among the most significantly downregulated miRNAs in trastuzumab-resistant breast cancer cells, which is attributed to epigenetic mechanisms involving DNA methylation and histone deacetylation. Restoring cellular miR-375 level suppresses trastuzumab resistance of breast cancers by directly targeting the insulin-like growth factor 1 receptor (IGF1R). Our study suggests the therapeutic potential of miR-375 for HER2-positive breast cancers in combination with trastuzumab.

## Abbreviations

HER2: Human epidermal growth factor receptor 2; IGF1R: Insulin-like growth factor 1 receptor; UTR: Untranslated region; TSA: Trichostatin A; 5-Aza-CdR: 5-Aza-2′-deoxycytidine.

## Competing interests

The authors declare that they have no competing interests.

## Authors’ contributions

XMY, HYZ, WDB and LW acquired and analyzed data. TW and YC analyzed data. AGY conceived and designed studies. LTJ conceived and designed studies and wrote the manuscript. All authors read and approved the final manuscript.

## Pre-publication history

The pre-publication history for this paper can be accessed here:

http://www.biomedcentral.com/1471-2407/14/134/prepub
